# Gold-Induced Cytokine (GOLDIC®) Therapy in the Management of Knee Osteoarthritis: An Observational Study

**DOI:** 10.7759/cureus.46231

**Published:** 2023-09-29

**Authors:** Sharmila Tulpule, Madhan Jeyaraman, Tarun Jayakumar, Naveen Jeyaraman, Asawari Bapat, Sankalp Yadav

**Affiliations:** 1 Orthopaedics and Regenerative Medicine, Orthobiologix Clinic, Mumbai, IND; 2 Orthopaedics, ACS Medical College and Hospital, Dr MGR Educational and Research Institute, Chennai, IND; 3 Orthopaedics, KIMS-Sunshine Hospital, Hyderabad, IND; 4 Clinical Pathology, Infohealth FZE, Dubai, ARE; 5 Internal Medicine, Shri Madan Lal Khurana Chest Clinic, New Delhi, IND

**Keywords:** osteoarthritis, gelsolin, knee, cytokines, gold

## Abstract

Background: Current treatment modalities for knee osteoarthritis (OA) provide symptomatic cures rather than reversing the pathology in the long term. An innovative regenerative therapy called "Gold Induced Cytokines" (GOLDIC®) was explored in various musculoskeletal diseases such as knee OA, lumbar canal stenosis, Achilles tendinopathy, and plantar fasciitis. In this study, we explored the safety and functional outcome of GOLDIC® injections in knee OA (KL grades 3 and 4) with visual analog scale (VAS) and Western Ontario and McMaster Universities Osteoarthritis Index (WOMAC) scores.

Materials and methods: A multi-center open-label observational study was carried out after screening the cases according to the inclusion criteria. A total of 106 knees in 65 patients were enrolled for four doses of 4 ml of ultrasound-guided intra-articular GOLDIC® injections every three to six days. All cases were followed up with pre- and post-VAS and WOMAC scores at an interval of four weeks, three months, six months, and one year, and the complications (including severe adverse reactions) were monitored throughout.

Results: In this study, 66.1% had grade 4 OA knee (without gross varus or subluxation) and 33.8% had grade 3 OA knee. All the participants underwent the GOLDIC® treatment modality. A statistically significant difference was observed in pre- and post-procedural follow-up in VAS and WOMAC scores at one-year follow-up. There were no recorded severe adverse reactions during the entire study period. Three patients failed the treatment in one year.

Conclusion: The GOLDIC® procedure shows great promise as a novel method for treating moderate to severe OA of the knee, both in terms of pain and functional outcome without any severe adverse reactions, in a sustained manner and is worth exploring as a long-term treatment option.

## Introduction

Knee osteoarthritis (OA) is a widespread and debilitating ailment affecting millions of people worldwide [1]. Articular cartilage is characterized by a limited ability to heal naturally due to its lack of blood vessels, undifferentiated cells, and the presence of specialized cells with limited mitotic activity. This leads to the progression of articular cartilage degeneration, synovial inflammation, and subchondral bone remodeling. Consequently, individuals experience stiffness, pain, and functional limitations, which significantly diminish their quality of life [2,3]. Due to increased longevity, the incidence of OA is bound to increase in the coming decades [4]. Current treatments for knee OA include pharmacological, nonpharmacological, and surgical approaches, but none of these can regenerate lost cartilage or change the course of the illness [5]. When grade 3 or 4 OA is present, total knee replacement (TKR) is typically the preferred treatment option when conservative measures have proven ineffective. However, it's important to note that TKRs are invasive procedures that can carry various direct complications and other related health issues. In rare cases, they can even result in mortality.

Because of the growing demand for non-surgical approaches to managing primary knee OA, there is a rising trend in the use of biologics such as platelet-rich plasma (PRP), mesenchymal stem cells, conditioned autologous serum, and growth factors in the field of regenerative orthopedics [6,7]. Intra-articular injections, including hyaluronic acid (HA) and corticosteroids, have shown effective short-term pain relief but do not influence the progression of the disease over time.

GOLDIC® or "Gold Induced Cytokines" refers to an innovative regenerative therapy that uses conditioned serum rich in cytokines (Gold-IC) by utilizing specialized gold particles that react with the patient’s own blood. Gold compounds, such as aurothiomalate, have been found to inhibit the production of nitric oxide (NO) by chondrocytes. Nitric oxide plays a role in mediating the destructive effects of interleukin-1 (IL-1) and tumor necrosis factor-alpha (TNF-α) in the joint. These effects include a reduction in collagen and proteoglycan production, the apoptosis (cell death) of chondrocytes, and the stimulation of matrix metalloproteases. In vitro studies have demonstrated that when cells are exposed to gold particles, they exhibit inhibitory effects on catabolic factors, an increase in both anticatabolic and anabolic factors, and an elevation in the levels of gelsolin. Gelsolin is a key protein involved in cellular metabolism, and its increased presence suggests a potential positive impact of gold compounds on cellular processes within the joint. Although many studies have demonstrated promising early clinical results in various degenerative diseases in animals, the conclusive benefit of GOLDIC® therapy in humans is yet to be seen for its implementation in clinical practice.

The primary aim of this study was to assess the safety and functional outcomes of patients who underwent gold-induced cytokine (GOLDIC®) therapy for OA of the knee using the Western Ontario and McMaster Universities Osteoarthritis Index (WOMAC) score. Secondary objectives included assessment of the global rating of change among these patients, the visual analog scale (VAS) score, patient satisfaction, and complications following the procedure.

## Materials and methods

Following the receipt of ethical clearance from the independent ethics committee at Orthobiologix Clinic in Mumbai on January 12, 2020, a multi-center open-label prospective study was conducted. The aim of this study was to evaluate the effectiveness and safety of autologous intra-articular GOLDIC® therapy in patients diagnosed with moderate to severe knee OA. All patients who participated in the study provided written informed consent.

A total of 89 consecutive patients, corresponding to 136 knees, were initially considered for the investigation. Subsequently, after applying inclusion and exclusion criteria, 65 patients and 106 knees were enrolled in the study between the years 2020 and 2022 in Dubai, UAE and Mumbai, India. The patients were followed up for a period of 12 months to assess the outcomes of the treatment.

Patients who were more than 18 years of age, patients with knee pain lasting more than six months, patients who underwent conservative treatment with no relief, and patients who were radiologically diagnosed with Kellgren Lawrence (KL) grade III and IV knee OA were included in the study.

Patients who are less than 18 years of age, patients who are radiologically diagnosed with KL grade I and II knee OA, patients with mechanically deranged joints (gross varus/subluxation), patients with stiff knees (fixed flexion deformity/limited range of movement), patients with neurological disease, inflammatory or autoimmune arthritis, secondary arthritis of the knee, patients who had taken any form of intra-articular injection three months before the study protocol, patients with active infection, immunosuppression, bleeding disorder, pregnant women and lactating women, and patients with malignancy were excluded from the study.

**Figure 1:**
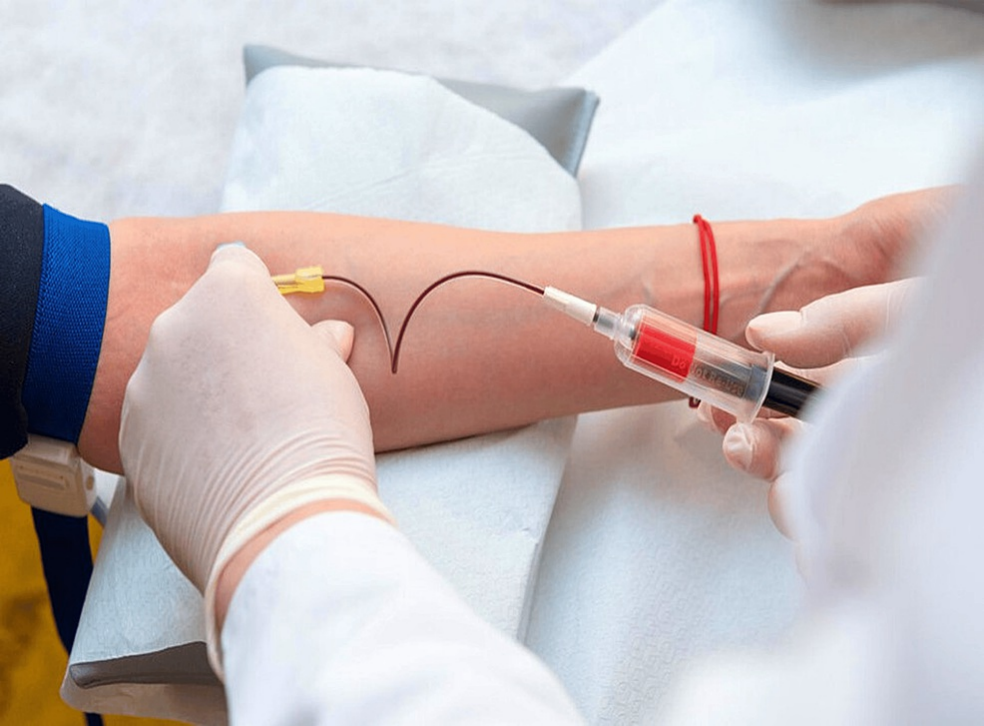
Withdrawal of peripheral venous blood from the cubital vein in GOLDIC® BTS syringes (Arthrogen GmbH, Ringsee, Germany) Picture courtesy of Dr. Sharmila Tulpule

**Figure 2:**
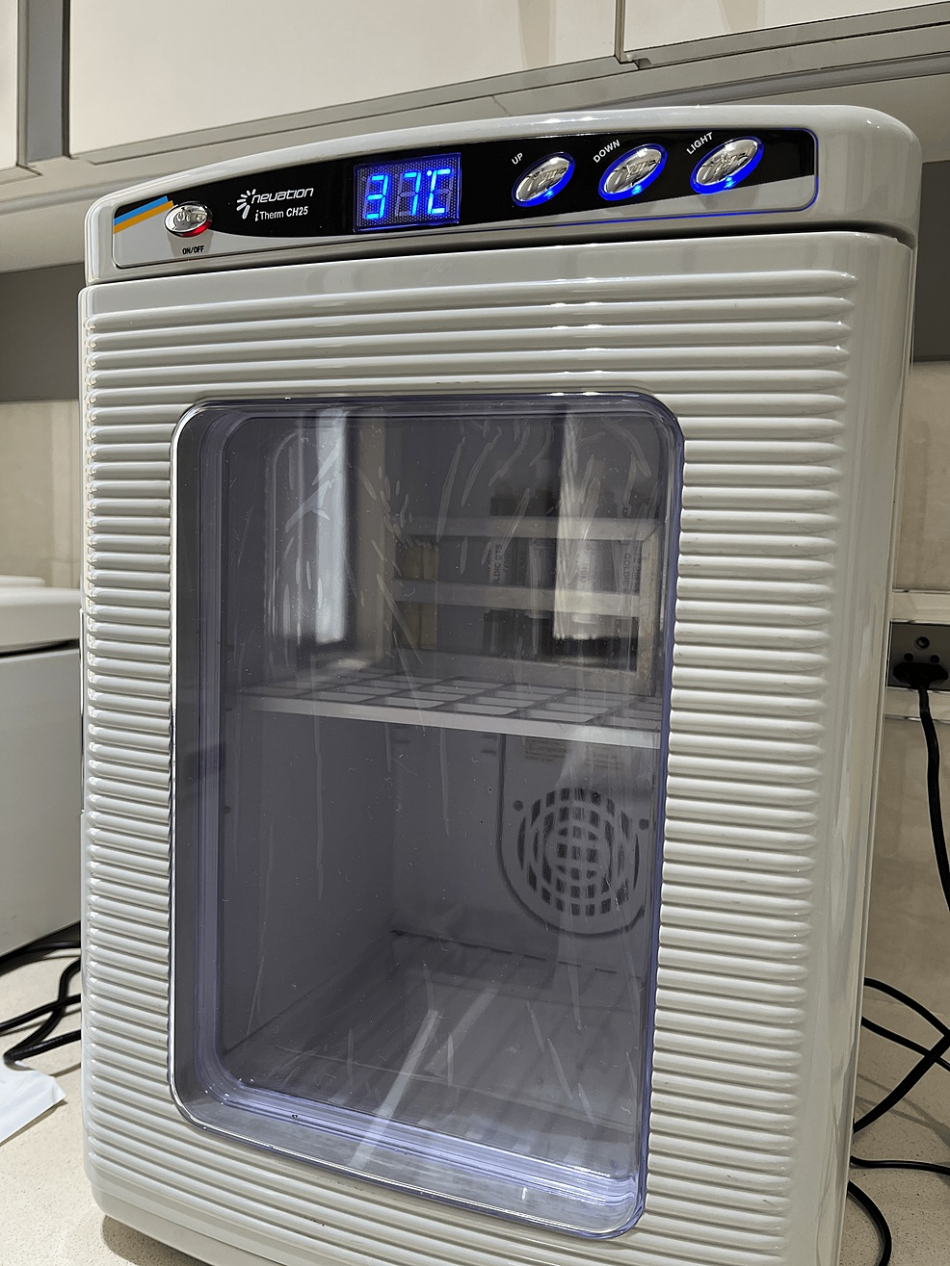
Incubator for GOLDIC® BTS syringes (Arthrogen. GmbH, Ringsee, Germany) at 37°C for 24 hours Picture courtesy of Dr. Sharmila Tulpule

**Figure 3:**
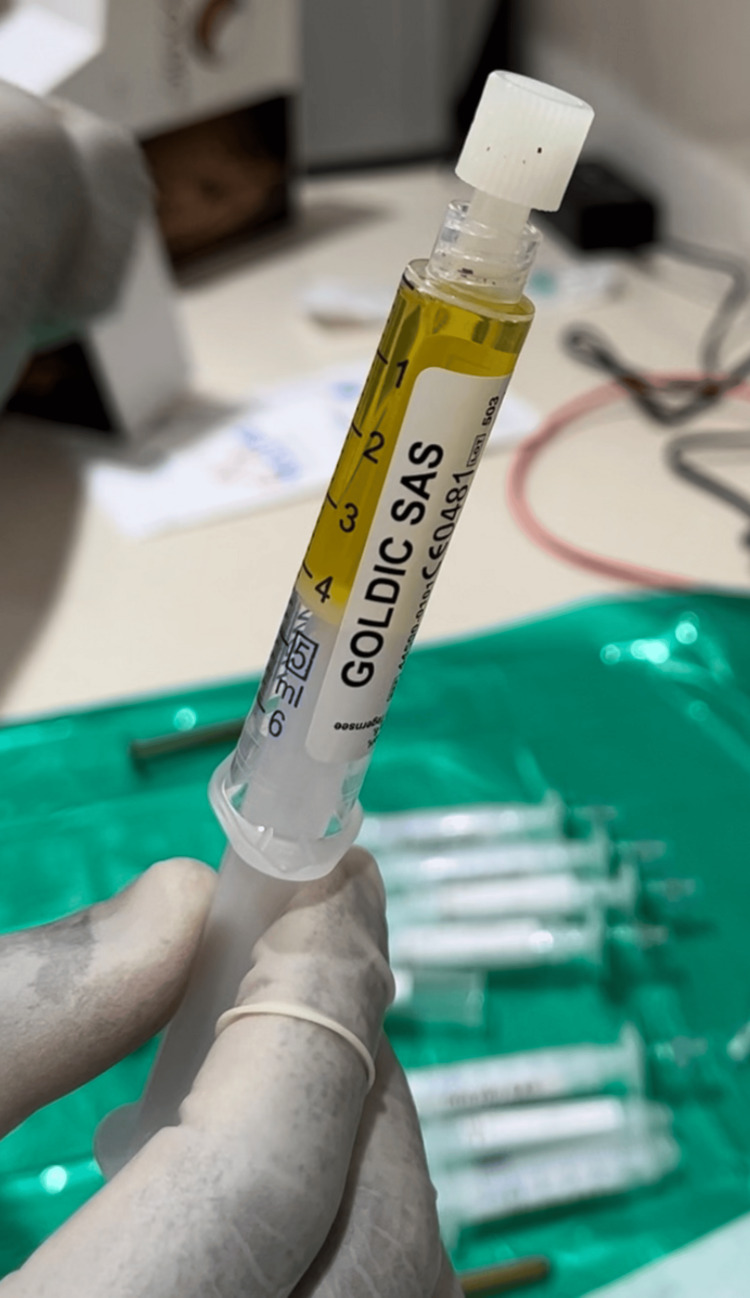
Resultant serum after centrifugation at 4000 rpm for 10 minutes Picture courtesy of Dr. Sharmila Tulpule

**Figure 4:**
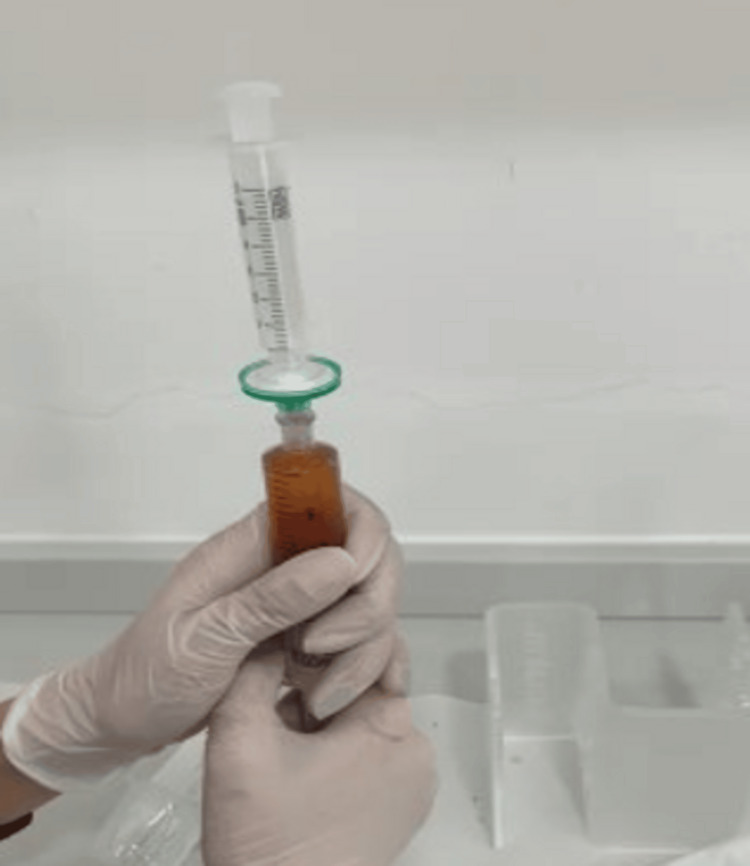
Filtration of supernatant-conditioned serum through a 0.22-μM syringe tip filter Picture courtesy of Dr. Sharmila Tulpule

**Figure 5:**
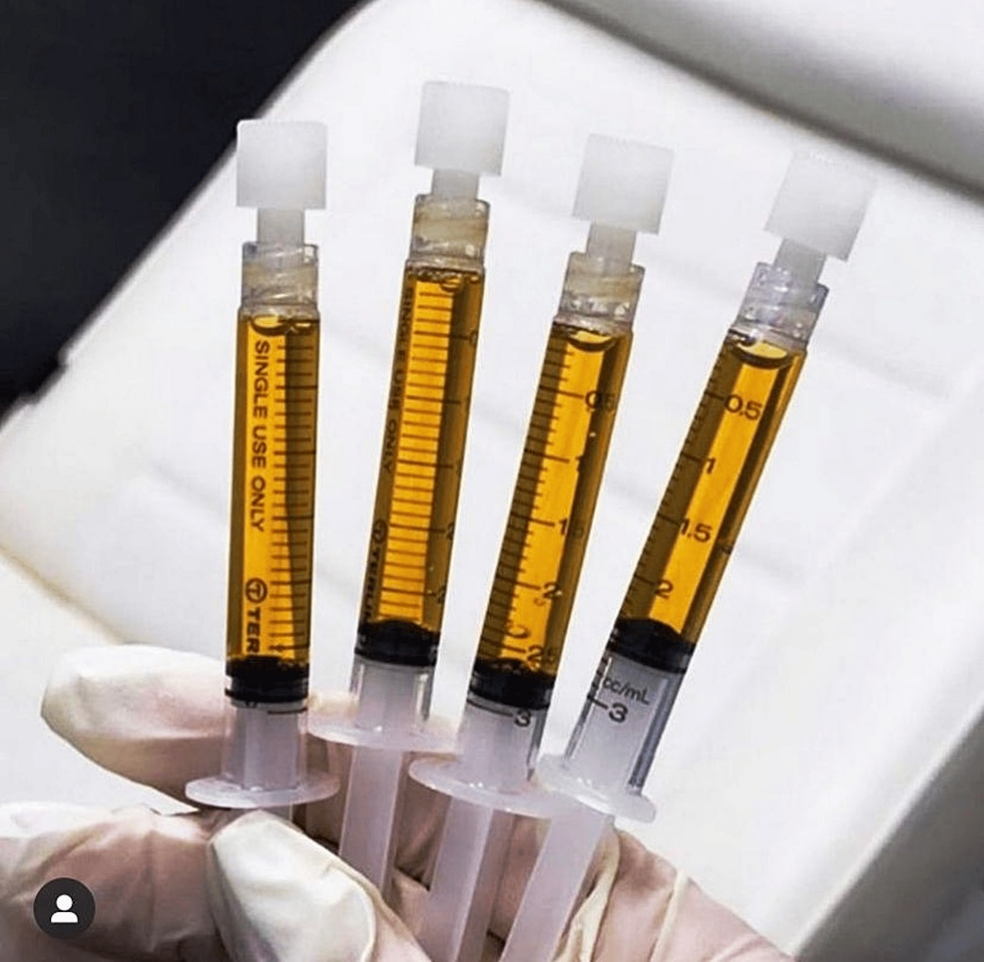
Final GOLDIC® injectate Picture courtesy of Dr. Sharmila Tulpule

GOLDIC® preparation: For GOLDIC® therapy, each patient had 4 × 10 mL of blood collected using four GOLDIC® BTS syringes (Arthrogen GmbH, Ringsee, Germany) at the start of the therapy. These four syringes were then placed in an incubator set at 37°C for a period of 24 hours. Following the incubation process, the contents of the four tubes were subjected to centrifugation at 4000 rpm (equivalent to 2250g) for 10 minutes. The resulting supernatant, which is the conditioned serum, was carefully collected and passed through a 0.22-μM syringe tip filter (Millex GP, Merck Millipore, Cork, Ireland) (Figures 1-5).

This filtered, conditioned serum was subsequently utilized for immediate intra-articular (IA) injection, or it could be stored at a temperature of -20°C for future use for up to eight weeks from the time of processing.

All patients underwent a treatment regimen involving four IA injections of approximately 4 ml of GOLDIC® serum. These injections were administered at intervals of three to six days. Each injection was performed under ultrasound guidance using a superolateral approach, ensuring strict adherence to aseptic techniques. A 22G spinal needle was utilized for the injections. In cases where patients had knee effusion, synovial fluid was aspirated under sterile conditions, and the amount of aspirated fluid was recorded. The aspirated fluid was then sent to an independent laboratory for microscopy to rule out any potential infection.

Patients were advised to manage knee pain at home by using ice packs or cold therapy and were instructed to avoid prolonged walking and standing for a period of 24 hours following each injection. Additionally, patients were specifically instructed to use only paracetamol for any post-injection pain and were cautioned against using non-steroidal anti-inflammatory drugs (NSAIDs).

After the injections, patients were encouraged to engage in post-injection rehabilitation. They were advised to abstain from strenuous exercise and were only allowed to participate in non-impact activities like walking, cycling, and pool exercises. Gradual resumption of normal sports or recreational activities was permitted after a certain recovery period. While not mandatory, supervised physiotherapy was recommended for most patients. All patients were followed up with VAS and WOMAC scores pre-procedurally and post-procedurally at regular time intervals of 3, 6, 9, and 12 months. The complications were also noted during the study period.

Statistical analysis

Descriptive statistics were used to summarize the data, with continuous variables presented as mean values along with their standard deviations (SD), while categorical variables were presented as frequencies and percentages. To assess the significance of changes within the same group over time, a repeated measures analysis of variance (ANOVA) was employed. The statistical analysis was carried out using IBM SPSS Statistics for Windows, Version 26.0 (IBM Corp., Armonk, NY).

## Results

The mean age of the study participants was 63.82 ± 11.36 years. Most of the study participants were in the age group of 51-60 years (32.3%), followed by 61-70 years (27.7%), 71-80 years (26.2%), 41-50 years (6.2%), 81-90 years (4.6%), and 30-40 years (3.1%). There was a female preponderance among the study participants. About 61.5% had both knees affected, 18.5% had their left knee affected, and 16.9% had their right knee affected. In this study, 66.1% had a Grade 4 OA knee and 33.8% had a Grade 3 OA knee. All the participants underwent the GOLDIC® treatment modality. Around 84.6% had no complaints, 10.7% had mild pain, 3.08% had mild effusion, and 1.54% had transient pain on the same day (Table 1).

**Table 1:** Distribution of demographic details among the study participants

Variables	Frequency	Percentage
Age (years)		
30-40	2	3.1
41-50	4	6.2
51-60	21	32.3
61-70	18	27.7
71-80	17	26.2
81-90	3	4.6
Gender		
Male	17	26.2
Female	48	73.8
Diagnosis		
Both knee	41	63
Left knee	12	18.5
Right knee	12	18.5
Grade		
Grade 3	22	33.8
Grade 4	43	66.1
Adverse reactions		
No complaints	55	84.6
Mild pain	7	10.7
Mild effusion	2	3.08
Transient pain on same day	1	1.54

There was a significant effect of time on VAS score, F (1.188, 64) = 548.06, P <0.001 (Table 2).

**Table 2:** Distribution of VAS score among the study participants VAS: Visual analog scale

VAS	Mean	Standard deviation	P-value
Pre-injection	6.31	1.74	-
3 months	3.09	1.18	<0.001
6 months	2.54	1.17	<0.001
9 months	1.65	1.44	<0.001
1 year	0.43	0.61	<0.001

There was a significant effect of time on WOMAC score, F (8.108, 28) = 180.41, P <0.001 (Table 3).

**Table 3:** Distribution of WOMAC score among the study participants WOMAC: Western Ontario and McMaster Universities Arthritis Index

WOMAC	Mean	Standard deviation	P-value
Pre-injection	20.12	5.01	-
3 months	37.03	6.17	<0.001
6 months	46.62	10.55	<0.001
9 months	62.19	11.52	<0.001
1 year	76.27	18.21	<0.001

**Figure 6:**
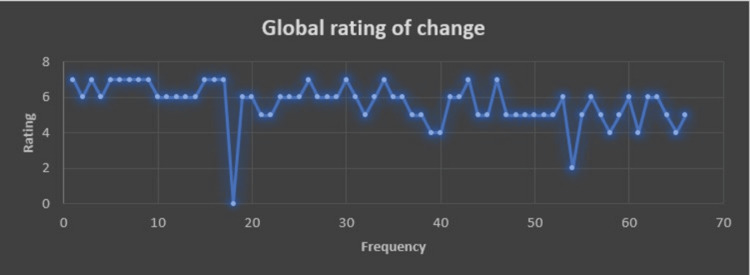
Global rating of change among the study participants

The mean global rating of the change in GOLDIC® therapy in knee OA is 5.75±1.01; range: 2-7 (Figure 6).

## Discussion

This study demonstrated the potential benefits of GOLDIC® therapy for the OA knee. The majority of patients reported a statistically significant improvement in the WOMAC score at each consecutive follow-up with a significant reduction in pain. No major complications were reported following the procedure, and most of the patients (n = 65, 106 knees) were willing to recommend GOLDIC® to others.

Cytokines, which are a group of peptides, primarily initiate or regulate the growth, proliferation, and differentiation of specific target cells. The significance of cytokines is steadily increasing in the fields of medicine and cellular biology [8]. The precise mechanism of action of the GOLDIC® treatment is unknown [9,10]. Following each injection of GOLDIC®, in vitro studies have demonstrated a notable rise in plasma gelsolin levels within the autologous serum as well as an increase in gelsolin levels within the synovial fluid [11]. Both gelsolin and granulocyte colony-stimulating factor (G-CSF) have been shown to have the capacity to stimulate regeneration [12]. In contrast to other blood-based biological methods, the GOLDIC® procedure stands out for its ability to upregulate both plasma gelsolin (pGSN) and G-CSF. These two components are significant because they play vital roles in tissue regeneration. Gelsolin is an actin-binding protein present in both the cytoskeleton and the plasma [13]. Indeed, the cytoskeleton plays a crucial role in determining cell viscoelasticity. Additionally, gelsolin influences various essential cellular functions, including cell motility, phagocytosis, and thrombocyte activation. Importantly, reduced plasma gelsolin concentration has been observed in degenerative tissue disorders, highlighting its potential relevance to such conditions [14,15]. Plasma gelsolin serves as a protective barrier to halt inflammatory responses within the body and has been observed to decrease during inflammatory arthritis, such as rheumatoid arthritis (RA) [16,17]. Interestingly, the gelsolin level was shown to be lower in the afflicted joints compared to the plasma level. As a result, it is remarkable that intraarticular therapy with gold-activated serum (GOLDIC®) resulted in a considerable rise in gelsolin in synovial fluid, making it a logical option for the management of OA [11]. Gold compounds (aurothiomalate) also limit chondrocyte nitric oxide (NO) synthesis, which is known to mediate the damaging effects of IL-1 and TNF-α, which include decreased collagen and proteoglycan formation, chondrocyte death, and stimulation of matrix metalloproteases [18].

Schneider et al. carried out one of the first phase 2a trials to evaluate the safety and efficacy of GOLDIC® therapy in the treatment of OA knee [11]. They reported significant improvements in both the Knee Injury and Osteoarthritis Outcome Score (KOOS) and WOMAC ratings at each follow-up, which is consistent with our findings. They also proved the efficacy of GOLDIC® not only in the management of Grade 2-3 KL KOA but also in patients with end-stage Grade 4 KL knee OA, as seen in our study. Other parameters, such as the age of the patient, gender, and body mass index (BMI) did not seem to affect treatment efficacy, and no major complications were reported with its use. Diffuse swelling and mild pain for the initial few days were the only reported complications following the procedure, which only lasted for two to three days. Plasma gelsolin concentration during the follow-up was also shown to have significant improvement immediately following the first injection. Unlike other blood-based product therapies such as PRP, bone marrow aspirate concentrate (BMAC), and autologous conditioned serum (ACS), which offer only temporary or short-term benefits, GOLDIC® therapy showed significant improvement in functional scores up to four years following the procedure, which could be mainly attributed to the increase in plasma gelsolin level and G-CSF, which are known to influence tissue regeneration and repair [15,19-22].

Some concerns have been raised about the use of gold-induced autologous cytokines in muscle diseases in studies on animals, including the possibility of possible heart failure in post-myocardial infarction patients. However, this study also found an increase in angiogenesis, myofibroblast proliferation, and collagen formation in the GOLDIC® group, which should be interpreted with caution [9].

This study's primary strengths lie in its prospective design, rigorous patient selection criteria, which resulted in zero loss to follow-up, and meticulous adherence to the study protocol. Conversely, the study faces significant limitations, including limited sample size (a larger sample would provide more robust scientific evidence on the usage of GOLDIC® in OA knee), a brief follow-up period (longer follow-up duration would enhance the study's robustness), limited adverse reactions data (since there were no severe adverse reactions, detailed information on any adverse reactions, their nature, and frequency is lacking, which is critical for evaluating safety), the absence of a control group (comparison, making it challenging to establish a direct link between GOLDIC® therapy and observed improvements), and outcome measures (the study uses VAS and WOMAC scores to assess outcomes, incorporating a broader range of outcome measures, including objective measures like imaging findings, could provide a more comprehensive evaluation). Consequently, it remains challenging to arrive at conclusive judgments regarding the effectiveness of GOLDIC® therapy. Nonetheless, the study does yield encouraging preliminary outcomes, underscoring the necessity for additional research to validate these findings.

Further high-quality randomized controlled trials, accompanied by extended follow-up periods, are essential to establish both the safety and effectiveness of GOLDIC® therapy before it can be routinely advocated for the routine clinical management of knee OA. Future research endeavors should also encompass a diverse range of objective metrics, including patient-reported outcome measures (PROMs), the correlation between PROMs and the patient-acceptable symptom state (PASS), as well as qualitative and quantitative magnetic resonance imaging findings. These comprehensive assessments will facilitate a more comprehensive evaluation of treatment outcomes and enable a more thorough cost-benefit analysis.

## Conclusions

The GOLDIC® procedure shows great promise as a novel method for treating moderate to severe OA of the knee, both in terms of pain and functional outcome. While the initial clinical results appear promising, it remains imperative to undertake extensive large-scale randomized controlled trials with extended follow-up periods to validate its true potential in comparison to other blood-based platforms. This will definitely be a remarkable step in pain management.
