# Correction: Gold-Induced Cytokine (GOLDIC®) Therapy in the Management of Knee Osteoarthritis: An Observational Study

**DOI:** 10.7759/cureus.c136

**Published:** 2023-10-09

**Authors:** Sharmila Tulpule, Madhan Jeyaraman, Tarun Jayakumar, Naveen Jeyaraman, Asawari Bapat, Sankalp Yadav

**Affiliations:** 1 Orthopaedics and Regenerative Medicine, Orthobiologix Clinic, Mumbai, IND; 2 Orthopaedics, ACS Medical College and Hospital, Dr MGR Educational and Research Institute, Chennai, IND; 3 Orthopaedics, KIMS-Sunshine Hospital, Hyderabad, IND; 4 Clinical Pathology, Infohealth FZE, Dubai, ARE; 5 Internal Medicine, Shri Madan Lal Khurana Chest Clinic, New Delhi, IND

This article has been corrected due to the published version containing multiple inaccuracies related to the study location and type. The published article originally and incorrectly described this is a single-center clinical trial taking place in Mumbai, when it was in fact a multi-center open-lable prospective study conducted in Dubai and Mumbai. Several instances of these descriptions have been revised for accuracy. The authors and journal regret these errors were not identified and corrected prior to publication.

