# Correction: Gold-Induced Cytokine (GOLDIC®) Therapy in the Management of Knee Osteoarthritis: An Observational Study

**DOI:** 10.7759/cureus.c138

**Published:** 2023-10-13

**Authors:** Sharmila Tulpule, Madhan Jeyaraman, Tarun Jayakumar, Naveen Jeyaraman, Asawari Bapat, Sankalp Yadav

**Affiliations:** 1 Orthopaedics and Regenerative Medicine, Orthobiologix Clinic, Mumbai, IND; 2 Orthopaedics, ACS Medical College and Hospital, Dr MGR Educational and Research Institute, Chennai, IND; 3 Orthopaedics, KIMS-Sunshine Hospital, Hyderabad, IND; 4 Clinical Pathology, Infohealth FZE, Dubai, ARE; 5 Internal Medicine, Shri Madan Lal Khurana Chest Clinic, New Delhi, IND

This article has been further corrected to update the conflict of interest disclosures with information pertaining to Dr. Asawari Bapat and Dr. Sharmila Tulpule's professional relationship with Orthobiologix Clinic, which offers the GOLDIC therapy treatment to patients.

